# Automated water recycle (AWR) method for dust removal from rooftop photovoltaic (PV) at Johor, Malaysia

**DOI:** 10.1007/s42452-022-05205-7

**Published:** 2022-10-31

**Authors:** Syed Zahurul Islam, Nur Syahirah Izzati, Mohd Noor Abdullah, Muhammad Saufi Kamarudin, Rosli Omar, Jasim Uddin

**Affiliations:** 1 Faculty of Electrical & Electronic Engineering, Universiti Tun Hussein Onn Malaysia(UTHM), Parit Raja, 86400 Batu Pahat, Johor Malaysia; 2 Department of Applied Computing and Engineering, Cardiff School of Technologies, Cardiff Metropolitan University, Western Avenue, Cardiff, CF5 2YB UK

**Keywords:** Photovoltaic cleaning, Dust-fall, Energy yield, Rooftop photovoltaic

## Abstract

**Abstract:**

Wet dust on the Photovoltaic (PV) surface is a persistent problem that is merely considered for rooftop based PV cleaning under a high humid climate like Malaysia. This paper proposes an Automated Water Recycle (AWR) method encompassing a water recycling unit for rooftop PV cleaning with the aim to enhance the electrical performance. This study makes a major contribution by developing a new model to correlate output power ($P_{out}$) and dust-fall factor. For model validation, we conducted an experiment of taking one set of Monocrystalline PV (mono) on a $340\frac{W}{m^{2}}$ of medium luminance day. One mono module was cleaned by AWR - pressurized water sprayed through 11 small holes over its front surface, while the other module was left with no-cleaning. The dust-contaminated water was filtered and collected back to the tank for recycling process. The water loss per cleaning cycle was achieved 0.32%, which was normalized to net loss of 28.8% at a frequency of 1 cycle/day for 90 days of operation. We observed that $P_{out}$ of no-cleaning PV was decreased by 29.44% than that of AWR method. From this experimental data also, a unique and more accurate model is created for $P_{out}$ prediction, which is much simpler compared to multivariables equation. Our investigation offers important insights into the accuracy of this regression model demonstrated by $R^{2}=0.744$ or a strong negative quadratic relationship between $P_{out}$ and dust-fall. The cleaning of PV modules is expected to save significant energy to reduce the payback period.

**Article Highlights:**

- An automated water recycle method for cleaning dust-fall in rooftop photovoltaic module is proposed.
- Both simulation and experimental models are developed to predict output power of the photovoltaic module.
- Proposed method can produce 24.40% more output power than a no-cleaning system with a mere water loss of 0.32%/cycle.

## Introduction

Dust adherence, mostly driven by wind, is a significant problem that impacts the performance execution, productivity, and energy output of photovoltaic (PV) panels in the context of Net Energy Metering (NEM) and large-scale solar generating. [1–3]. If the surface is not routinely cleaned, dust adhesion on the panel significantly effects on top layer, the dust surface can degrade the electromagnetic (EM) radiation absorption in the short circuit current and open circuit voltage by up to 22% and 6%, respectively [1, 2]. Malaysia has a lot of potential to use solar energy, but the country faces many challenges to harvest maximum power from PV generations. One of the major challenges is wet dust accumulation on the surface that causes a decline in output power and hence efficiency [3–6]. Moreover, low tilt angle, high humidity, low wind speed, bird droppings and permanent stains, and less rainfall cause high dust accumulation on the PV front surface. Due to the position of Malaysia near the equator, the climate of Malaysia is tropical with hot ambient temperature and has an uneven rainfall distribution throughout the year. Besides this, low wind ($<3\frac{m}{s}$) and high humidity (80-90% in rainy but 47% in dry season) are the common features in the climate of Malaysia [8]. Moreover, the 15^o^ tilt angle accumulates high dust-fall on the PV surface compared to higher inclined panels. The dust in Malaysia is acidic, wet, and can reduce output power up to 58.67% [3]. Thus, dust plays a significant role in the degradation of PV performance, resulting in reduced energy production, less energy sent to the grid, and economic loss in terms of grid payback.

There is a significant growth of solar energy capacity in Malaysia, from 179MW in 2018 to 725MW in 2019 [7], which is in line with the national goal to make RE 20% of the capacity mix by 2025 and becoming a low carbon economy. There are 3288 NEM customers exporting their surplus energy to the grid, a total of 265.27MW of power, according to TNB [8]. In the current NEM installation plan, there is a lack of integrated cleaning system. Therefore, a huge amount of electricity is wasted due to dust adhesion on the PV surface every year. This waste and poor performance of PV might discourage many future NEM consumers. Recently, many large solar generators with up to 22,000 panels (20,000$m^2$ of land space) maintain the cleaning by deploying automated or robotic devices [9]. However, this method is not feasible for rooftop PV application due to its cost and small capacity. As a result of dust adherence on the PV surface, a significant quantity of power is lost annually. This waste and poor performance may dissuade many potential Net Energy Metering (NEM) users.

In this study, we propose an Automated Water Recycle (AWR) method in conjunction with a water recycling system to perform wet dust-fall cleaning of the PV surface. In the simulation, we have calculated the dust-fall factor of a PV module and measured its output power reduction due to soiling on its surface. Then we have developed a mathematical model of output power versus dust-fall factor to determine the correlation between them. To analyze the effectiveness of the simulation model, we set up a testbed by manually spreading wet sand over its surface from 15g to gradually 75g between 8:00 AM to 6:00 PM. Simultaneously, the electrical performance of the AWR method and no-cleaning PV modules, namely output power, energy yield, and efficiency are evaluated during this time. We also analyzed the loss/cycle of the water recycling process to compare the effectiveness of the AWR method system with previous researchers. Based on the experimental data, we have developed another new and more accurate model compared to the simulation model through regression analysis. The outcome of the research can be seen as a support of the 12$^{\hbox {th}}$ Malaysia Plan (2021-25) development to speed up solar energy capacity in NEM and holistic and sustainable management of energy and water supply.

The novelty of this study is that we have developed two new models through regression analysis to predict the PV output power from the dust fall on its surface and investigated their degree of relationship under the climate of Malaysia, while obtaining the net loss of the water recycling process as a part of analyzing the effectiveness of the proposed AWR method deployed in rooftop PV application.

There has been a significant increase in dust studies since 2011, where almost half of it was conducted in Saudi Arabia, India, and Malaysia. The dust decomposition density within 30-45 days was recorded by the few researchers, such as (in $\frac{g}{m^2}$) 3.8 (Malaysia) [4], 4.6 (Pakistan) [10], 5 (July) to 28 (August and October) in Saudi Arabia [11], 5.12 ± 0.55 (Western India) [12], 31-127 (Qatar), 0.2-2.44 (Kuwait), and 4.48-15.84 (Egypt) [13]. In arid regions like Iran, less than 1km visibility was reported during the dust storm (March to May) [14]. It was found that energy loss due to dust decomposition in Monocrystalline PV (mono) type (16%) is more than other types of PV, such as polycrystalline or poly (11%) [10]. In contrast to [10], researchers from India argued for a higher power drop in poly (22.96%) than mono (20.4%) module [15]. In the same vein, Hai Jiang et al. found in China that mono is slower in efficiency degradation than poly against the same dust density [16]. The degradation caused by the PV type is still unclear. It might be one of the reasons for the most utilized PV module is found to be multicrystalline (37%), compared to that of mono type (27%) [17].

In recent years, there has been a rise in the quantity of literature about various PV cleaning methods. On the basis of our study of prior works, the PV cleaning techniques are categorised as preventative, automated, and manual. Manual PV cleaning requires hand-use and a long-stick brush. The automated technique involves a robotic or machine-integrated cleaning system, whereas the preventive method uses PV surface coating material. We discovered three techniques to automated cleaning: water-oriented cleaning, dry and wet cleaning, and non-contact cleaning. Previous research indicates how the tiny particles of dust can accumulate and deteriorate the PV cleaning output system. Some studies have scrutinized dust particles and its effect on PV performance degradation in their respective countries. Robot integrated systems are found infeasible for residential PV systems. Therefore, almost all the researchers have agreed on deployment of forced water or air techniques because it is the cheapest and effective way to clean the PV surface through automatic and manual methods for this application.

In [18], an automated residential water based cleaning system for 100W M36 mono PV panel was proposed. The system worked from 8.00 AM. till evening while a flashlight integrated scarecrow was added to scare the birds away from sitting on the panel and leaving their droppings. The system improved the current from 3.13A (with dust) to its manufacturer-rated maximum current 5.6A (after clean). Another water based own-designed cleaning robot was constructed using Arduino Uno, 24V DC motors(15W), batteries, and L298N H-Bridge boards and then tested for cleaning 1MW capacity of PV generation in Zahrani, Lebanon [19]. The process was undertaken early in the morning, which took around 4h to clean 54 PV strings. From the 105 days’ data collection, an increase of 32.27% in power was achieved for a multicrystalline PV module, installed at 9$$^{\hbox {o}}$$ tilt angle. The cost of the system was US$650.

A technique to reduce the water consumption was investigated by M Nateqi *et al.* at different frequencies (0.1, 0.2, and 0.33Hz) of spray water over the PV surface [20]. The analysis showed water spray for 0.2Hz or 5s could reduce consumption by 50% compared to the steady state spray method. A self-priming electric pump, controlled by a programmable logic controller (PLC) was used to lift water from a subterranean tank and then subsequently sprinkled the water over the PV surfaces [11]. The system provided 27% more power than the non-cleaning system by compromising 9L of water. Hussein A. Kazem et al. showed in their study in Oman that the PV efficiency was degraded by 12% after two months of installation for not cleaning it [21]. They conducted the experiment taking two-identical PV panels, solar radiation transmitter sensor, dual display, and a temperature sensor. They analyzed nine types of cleaning methods from where they concluded that the water could be sufficient to clean the PV panels. Taking 2 sets of mono and poly PV modules, Rizwan Majeed conducted a dust removal experiment using pressurized water to spray over the surfaces [10]. The process required an average of $1.8L/m^2$ of water and managed to recycle 55% of it. Overall the technique improved the mono and poly modules’ efficiency (98%) as well as output power (11.5 to 16%) immediately after the cleaning. F. Ekinci et al. compared performance three different kinds of chemical cleaners, namely 2-propanol, ethanol, acetone, and water based on the ability of natural dust cleaning of a 50W PV at Turkey [22]. They performed the cleaning service using a cleaning robot with fogging nozzles, ejection angle and pressure 1.4-7.8 L/min and 0.2-2.5MPa, respectively. Analyzing the I-V characteristics of the module, the researcher found output power enhancement for using the 4 cleaners, which were 15% (2-propanol), 14% (ethanol), 11% (acetone), and 10% (water). In the USA, a study on the soiling effect for PV was conducted by [23]. The study showed 7.4% loss in efficiency from an analysis of 145 summer days in several cities. In a drought city, the efficiency loss was around 14% for a 15% efficient module. The study recommended a robotic cleaning that could again have an annual energy yield of 9.8%.

B. Parrott utilised a sort of silicon rubber foam brush without water for dry cleaning [9]. The study was done in Saudi Arabia using 10kW of monotype, grid-connected PV generation placed at a tilt angle of 25$$^{\hbox {o}}$$. The 36kg robot was meant to move beside the PV module between 6:30 AM and 7:00 AM while it cleaned the surface with a 120 rpm brush speed. The data was gathered for 37 days between 11:00 a.m. and 1:00 p.m. with a resolution of 5 minutes. It demonstrated that frequent robotic cleaning was superior than monthly and weekly hand cleaning. Based on a normalised examination of the 1GW capacity of a PV production, the study revealed an annual power loss of $1 million if cleaning occurs every two weeks rather than daily. Xiaoqiang Du et al. analyzed fine dust particles on PV surfaces and analyzed the removal of dust particles through turbulent airflow in both dry and wet conditions [24]. They achieved 95.12 (dry) and 82.16% (wet) dust removal rates at 0.8 Mpa inlet pressure. The 9.0 mm diameter of the air inlet was designed at the middle of the outlet to create pressure all over the PV surface. Another research in a university in Northeast China recently developed a lightweight robot (8kg) that could do dry cleaning up to $$30^o$$ climbing angle. The research claimed an effective dust removal rate of 92.46% and increase PV efficiency from 11.06% to 49.53% [25].

**Table 1:** Summary structure of related previous works based on literature review

Year & Ref	Location and PV type, tilt angle	Cleaning type	Significant method and outcome	Different from this study
2018 [9]	Thuwal, Saudi Arabia mono (10kW, $$25^o$$)	Dry	-Robotic- silicon rubber foam brush with aluminum frame -Fortnight manual cleaning is 1.5% less effective than a daily robotic cleaning	Automated robotic system for large solar application, not suitable for NEM
2019 [21]	Oman, mono (125W, $$18-25^o$$)	-	-Dust accumulation analysis at 6 locations in Oman -Power production decrease by 55%	Limited to dust analysis and effects on power only
2019 [11]	Jeddah, Saudi Arabia mono (8kW, NA$$^{*}$$)	Non-contact and wet	-Vibration, air and water jets, and combinations of these. -Power output increasing by over 27%	Water loss per cleaning cycle is 9L, no recycling method was considered
2019 [24]	Zhejiang Sci-Tech University, China mono (short circuit current 8.78mA, NA$$^{*}$$)	Non-contact approach	- Turbulent airflow at 94m/s speed in both dry and wet condition -Design model of nozzle validates experiment -Dust removal rate 95.12 (dry) and 82.16% (wet) at 0.8Mpa pressure	-Power requirement for turbulent airflow was ignored. -Our study proposes water flow, different than airflow
2020 [10]	Islamabad, Pakistan mono and poly (40W each, $$15^o$$, $$34.5^o$$, $$60^o$$ )	Wet	-Flat-fan nozzles with pressurized water with recycle process, 2 months of data collection -Achieved 98% of efficiency within 35s of operation	Dust accumulation rate is different due to different climate zone and tilt angle
2019 [19]	Lebanon, multicrystalline (310W each, total 1MW, $$9^o$$)	Wet	Own-designed and manufactured robot, 105 days data collection, an increase of 32.27% in output power	Suitable for large solar generation. System cost (US$650) is too expensive for roof-top PV cleaning.
2022 [22]	Adana,Turkey, NA$$^{*}$$ ($$50W\times 4pax= 200W$$, NA$$^{*}$$)	Wet	-Robot is manufactured by 3D printer -Oneday data collection from using 3 alcoholic cleaners -2-propanol and water can enhance 15 and 10% in PV output power	-Alcoholic-based solutions are suited in winter countries to avoid freezing on PV surface. -Cost of the solutions/system was ignored.

$$^{*}$$Not Available

To the best of the authors’ knowledge, only a few researchers conducted the effect of dust accumulation and cleaning for roof-top PV in Malaysia. The study of the dust accumulation on the PV performance was carried out by S. A. Sulaiman et al. [4]. They found monthly degradation in the mono module is higher than the quarterly rate. The average dust density and power reduction were stated 2.5 $\frac{g}{m^2}$ and 0.5% (daily), 3 $\frac{g}{m^2}$ and 1.1% (weekly), 3.8 $\frac{g}{m^2}$ and 1.5% (monthly), respectively. Wan Juzaili Jamil et al. measured power loss against the soiling effect in Terengganu for 12 months [3]. They performed data collection at a 2.5MW PV site and observed power loss of an average of 26.22%, where the minimum and maximum losses were 4.86% and 58.67%, respectively. Conversely, Faridah Hanim et al. claimed 50% improvement in output power by using an Arudino integrated robotic cleaning system [5]. Similarly, a robot was used to clean poly type panel and claimed to obtain a 14.6% increase in voltage [6]. The details on the PV size, implementation, and performance analysis of these studies are insufficient.

Numerous studies have examined the effect of the automated cleaning method on the enhancement of the energy production of PV modules. As summarised in Table 1, the results of the studies conducted in various places have been published in the scientific literature. In Malaysia, there is no research on rooftop-based automatic PV cleaning systems and output power analysis, given what has been discussed in the literature that has been subjected to peer review. There remain several aspects of cleaning wet dust-fall for NEM application in Malaysia about which relatively little is known and where comprehensive research is lacking. Previous studies in Malaysia are limited to haze effect [26], glare analysis (simulation) [27], dust accumulation [4]. Researchers in Malaysia analyzed only dust adhesion without any cleaning system.

This issue must be taken into account both before and after the PV system is installed because it affects the owner, investor, or user’s return on investment. The purpose of this research is to enhance efficiency and mitigate power loss of the PV module by deploying the automated adhesion of dust-fall cleaning system or AWR for residential rooftop NEM application. Previous research indicates enhancement in efficiency is associated with high water consumption and pump capacity for increasing the water pressure on the PV surface. In this study, we have made an effort to minimize the water loss by using a recycling process. For this contribution, we have developed the AWR using a low-powered DC water pump, which is, controlled by an algorithm embedded in Arduino Uno along with a dust sensor. The system is then tested by building a real structure of two 10W mono PV modules installed at a 15° tilt angle in the premise of a solar site at Universiti Tun Hussein Onn Malaysia (UTHM). The AWR method also includes a water recycling and filtering unit to recycle and minimize water wastage. This study also simulated the output power of a selected PV module under the climate of Malaysia and quantified its yield reduction due to dust-fall factor through model analysis and experimental data observation. Further, this study developed mathematical models of output power versus dust-fall on PV surface to determine the correlation between them.

The organization of the paper is as follows. Section 2 explains the research trend on soiling and its scenario in Malaysia. Section 3 is explained by dividing into four subsections. First, the design of the AWR method is presented and then experimental setup is introduced. Second, method of dust collection and PV mounting systems are presented. Third, sizing of the water pump and tank and fourthly, filtration process is explained. Section 4 depicts the results and analysis, simulation, and mathematical model based on output power and dust accumulation. This section also analyzes the net water loss of the AWR method. Finally, Sect. 5 contains the conclusion and possible future directions for research.

## Analysis on PV Dust-fall in Malaysia

**Fig. 1:**
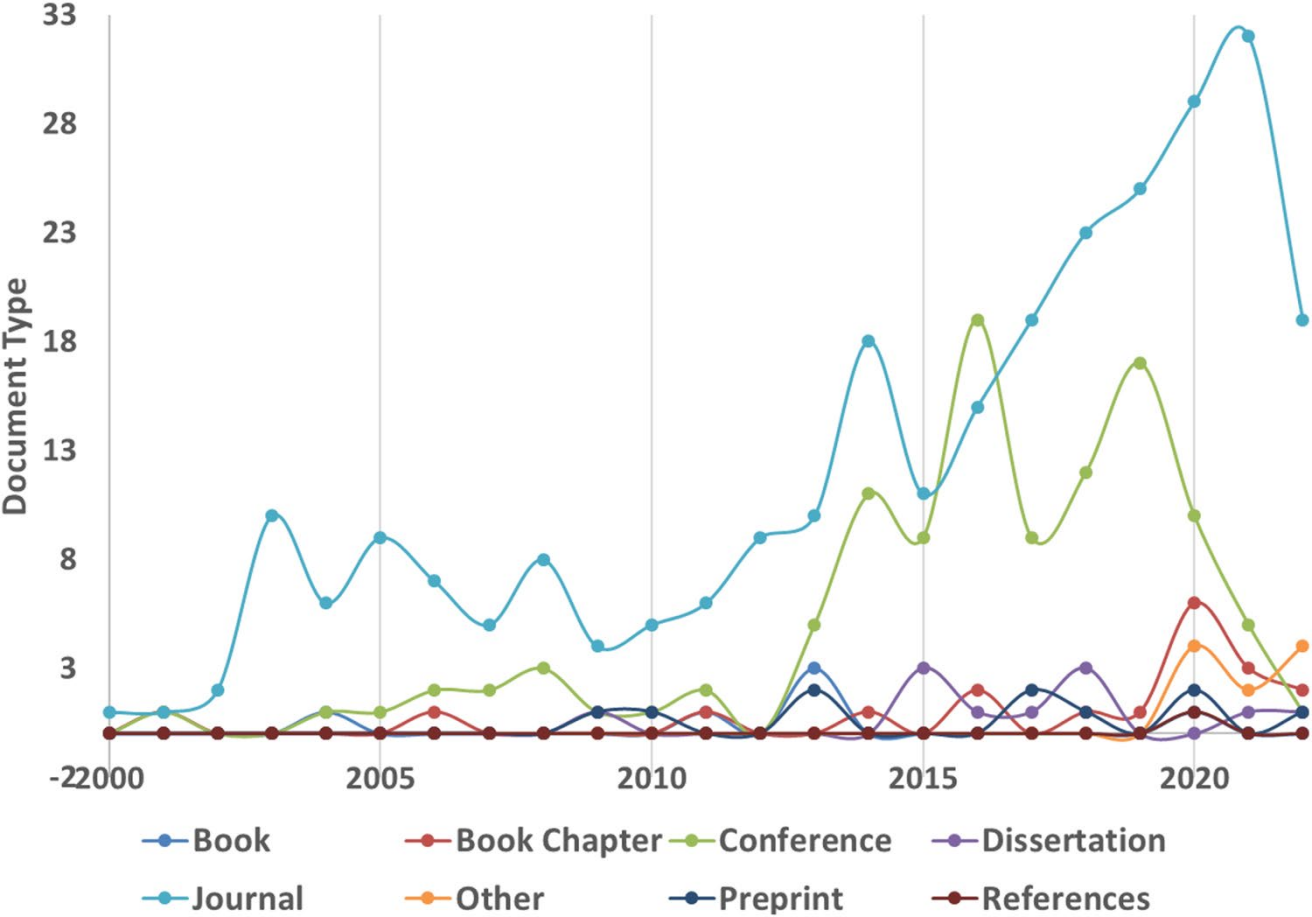
Trend of publication on dust-fall and soiling effect in PV since 2000. Data obtained from lens.org, search keyword: “solar soiling dust”

We collected records of publications on dust analysis from Lens.org and conducted a study on the number of publications. Figure 1 shows the number of documents that have been published since 2000 and investigated the effect of dust accumulation on PV panels. It can be observed from Fig. 1 that soiling was a concern prior to 2003, but was not a frequently published topic. This reason was the fact that the leading countries in the development of PV systems were mainly located in regions without dust problems. Since 2009 to date, the number of published documents has increased by almost 700%. Specifically, developing and installing PV systems in the Middle East and North Africa, the problems regarding the accumulation of dust have become more prominent. Therefore, a significant rise in the number of scientific studies has appeared, especially since 2009.

**Fig. 2:**
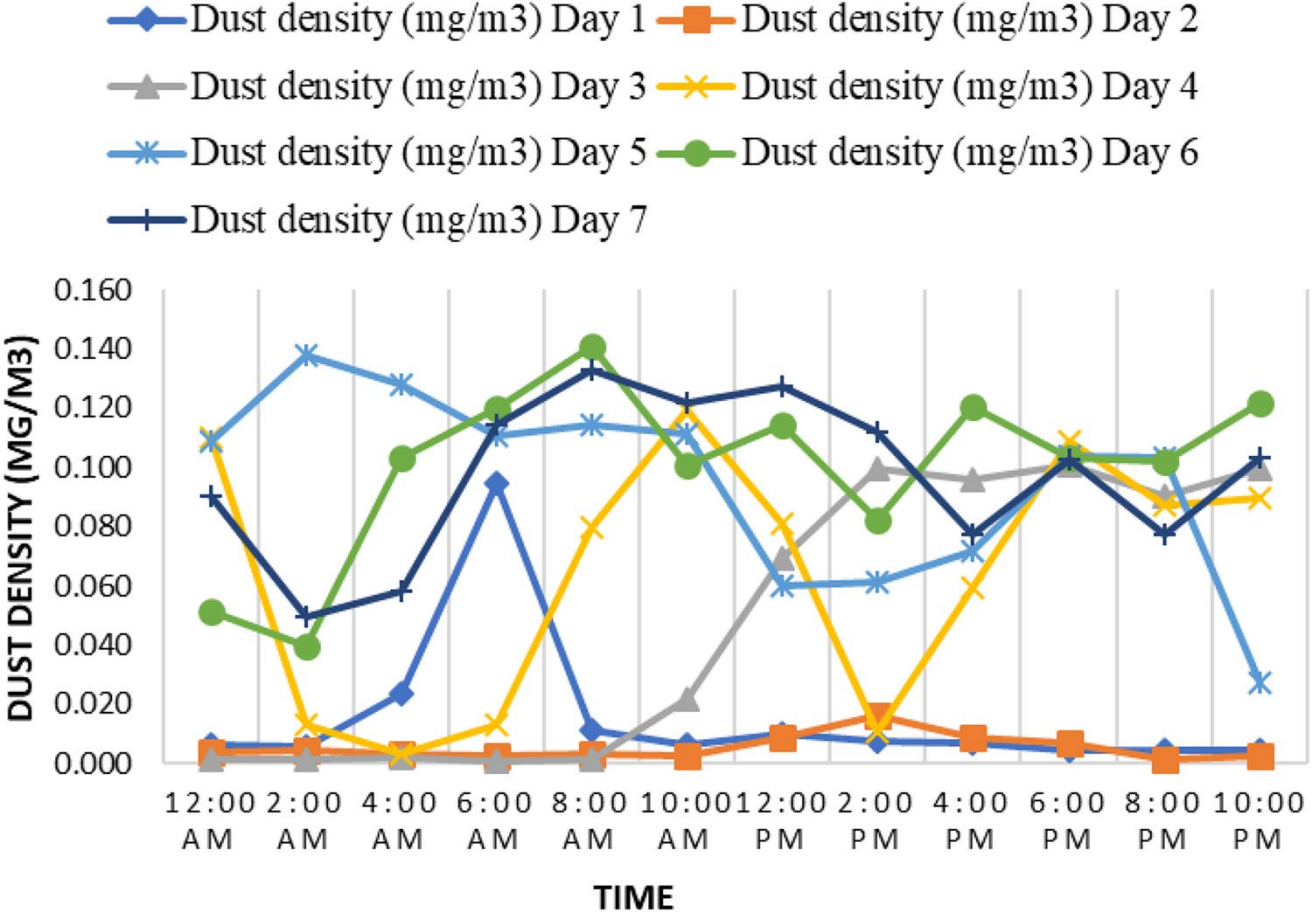
Dust density analysis for 7 days at UTHM, Johor, Malaysia

In addition to the statistical survey, we installed a dust sensor near the experimental location and recorded dust density (mg/m$^{3}$) for seven days. Figure 2 shows the recorded average of dust density collected at frequency of 1.0Hz. Due to the raining, the obtained dust density on Day1-Day3 days were found to be very insignificant. From Day4 to Day7, the weather was sunny and windy. This analysis shows that weather can affect dust density which might result in accumulation of wet dust on the PV module.

## Materials and methods

**Fig. 3:**
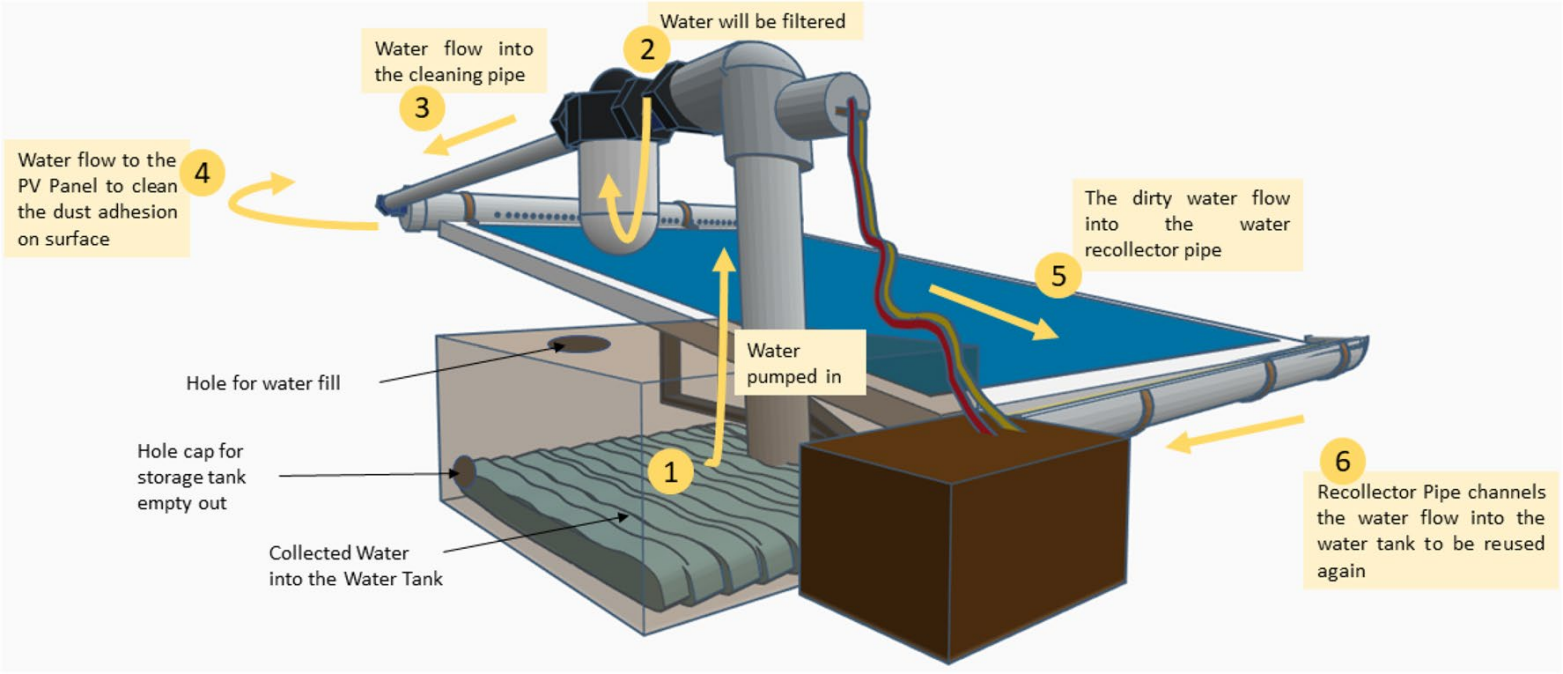
Conceptual 3D design of novel prototype—automated adhesion dust-fall cleaning or AWR method (used tinkercad.com application software)

This effort continues with the creation of a suitable experimental apparatus and method for carrying out the experiment. Before the experiment, a 3D conceptual design, circuit analysis, and algorithm for the automated system were created. Then, we proceeded to the experimental setup of the automatic water recycle system in order to examine the impact of wet sand on the energy production of two PV modules of identical construction. The experimental setup includes the installation of PV modules, dust collection, a PVC platform with an adjustable tilt angle, a 775 DC motor with a water tank, and a cleaning system. The 3D design of the novel prototype is shown in Fig. 3. The focus was on cleaning the dust adhesion on the PV module surface through pressurized water flow.

**Fig. 4:**
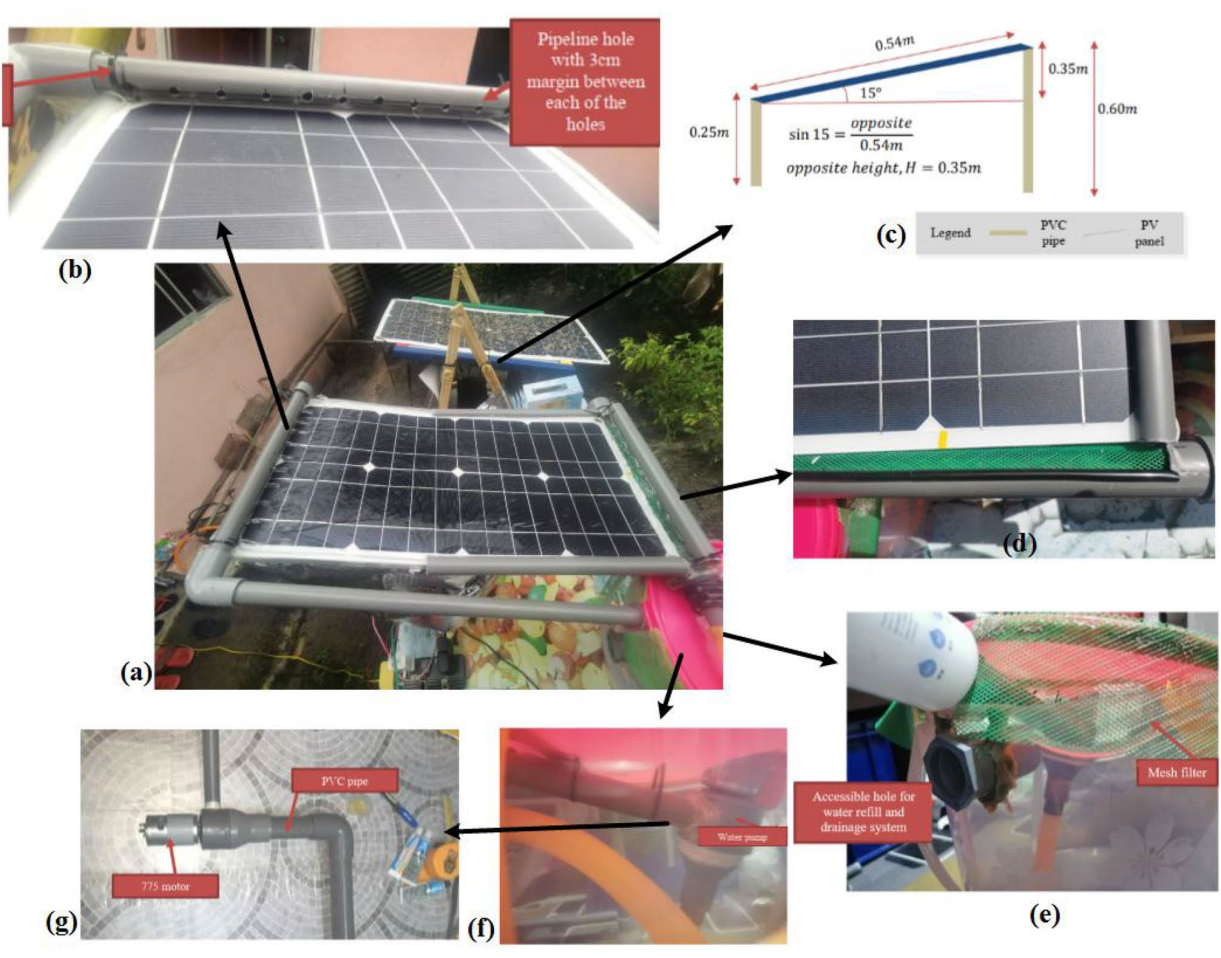
Experimental setup of PV cleaning prototype by developing a PVC and wood based mounting system at the rooftop of a specially designed room. (**a**) AWR method vs. no-cleaning system. Manual spread of wet sand over the PV surfaces (initial sand = 15 g). (**b**) PVC is clamped on top of the PV-stand using black cable ties and nylon zip. Pipeline hole at 5cm gap to spray water. (**c**) Trigonometric analysis for PV-stand design, both 10 W mono modules were installed at $15^o$ tilt angle facing to the north. (**d**) Mesh gutter to filter large debris after the water drains off cleaning. (**e**) Filter to purify the water before it is back to the tank. (**f**) 775 motor is installed as submerged in water. (**g**) Installation of motor coalesced PVC pipe

The design consists of a main pipe system to channel the water to the module, water filter, water reservoir and recycle chamber, and electrical parts - 775 Motor, Arduino Uno, optical dust sensor (Sharp GP2Y1010AU0F), and a DC relay. The experimental setup is shown in Fig. 4.

### PV modules setup

The outdoor experiments were conducted on the rooftop of a specially designed room located at a solar site in FKEE, UTHM. The data was collected on the day with a cloudy climate and almost the same solar irradiance. The capacity of the mono PV module was 10W which was installed at a 15^o^ tilt angle facing the northern hemisphere Fig. 4a–c. The type of PV used in this experiment is the mono module purchased online. Due to the limitation of the Covid-19 movement control order, the supplier was unable to supply the 100W PV module that was requested. The technical specification of the module is shown in Table 2. Developing an appropriate PV mounting system on a real rooftop, setting up a solar data logger, and collecting data for a longer period of time all presented challenges for the researchers. Nevertheless, a number of obstacles were surmounted, such as the development of a self-made mounting system utilising PVC-wood on a mock rooftop on university property.

**Table 2:** Electrical specification of the PV module

Type	Size, m	Voc, V	Isc, A	Vmpp, V	Impp, A	Pmax, W	$$\eta$$, %	Price, US$
Mono	0.54 × 0.28	21.4	0.53	20.0	0.5	10.0	6.6	37.00

The dust was manually spread over the PV surface at different measurements to study its effect on the output power. One set that contains two mono modules has a dimension of 0.54 m (L) $\times$ 0.28 m (W) $\times$ 0.025 m (T) and an area of 0.1512 $m^{2}$. One module was left dirty and the other one was cleaned (using AWR) from 8:00 AM to 6:00 PM. The output voltage and current at maximum power were recorded using a Multimeter.

### Dust collection, PVC stand, and tilt angle

The dust collection setup consisted of a PVC stand and a digital weight balance. The PVC stand was self-developed with a fixed tilt angle of 15^o^ as shown in Fig. 4c with its trigonometric analysis. The suggested angle was based on our previous research, suited for PV installation in Malaysia [28]. The opposite height of the stand was fixed at 0.60*m* to maintain the tilt angle and harvest maximum solar irradiation. Both PV modules were placed adjacent to each other, facing to the north. The dust sample consisted of wet sand and was collected in a glass plate under the same climatic disclosure in order to determine the dust density. Using a dust sensor installed adjacent to the PV system, the rate of dust deposition on a surface area was determined in terms of dust density. Prior to being physically dispersed across the PV surface and the dust sensor tunnel, the wet sand dust was weighed using a digital scale before being weighed again. The optical dust sensor (Sharp’s GP2Y1010AU0F) was installed next to the PV module to detect dust particles in air using its photo-sensor and infrared light emitting diode (7 days’ dust analysis was shown in Fig. 2). Since any debris could fall into the sensor hole and affect recording density data, we covered the hole using a mesh filter of 0.25m $\times$ 0.15m size to prevent entering debris into it. The sensor values were collected through serial monitoring from Arduino IDE. In this experiment, the dust density was fixed at 0.90 $\frac{\mu g}{m^{2}}$ (sensor shows 0.59 $\frac{\mu g}{m^{2}}$ at no dust condition). Figure 4a depicts the initial same amount of wet sand (15 g) spread evenly on the PV surface. To channeling water, we built the piping system using a fixed PVC pipeline structure. Prior to it, a thorough measurement was conducted according to the size of the PV module. Instead of using nozzles, we made 11 holes at a 3cm gap on the PVC pipe and placed them at the upper part of the PV module, as shown in Fig. 4b. The diameter of the hole was enough to create pressure and drain water on the surface up to 90%. A 15mm end cap was enclosed at the end of this PVC.

### 775 DC Motor and Water tank

A low-powered (12V, 18W), 5mm full round, and sufficient torque producing motor (774) was utilized as a water pump to create the pressurized water and channel from tank to upper end of the PV Fig. 4g. The motor was appropriate because the built-in fan provided high torque (stall torque 97Ncm at 14.4V) for this experiment. The appropriate size (98 $\times$ 42 mm) and weight (350g) of it was useful for these tasks. The motor was controlled by Arduino Uno together with a DC relay and dust sensor. The DC motor was powered by a 12V 1A adapter and switched ON/OFF when the threshold exceeded the dust density. The details of this algorithm is explained in Sect. 3.4. The motor was installed and submerged into a transparent water tank of 15 L capacity Fig. 4f, g. The collected recycled water was filtered by a specially designed mesh gutter of 39 cm length into the PVC pipe before it was sent back to the tank Fig. 4d, e. A 25 mm end cap was glued to this PVC pipe to direct the water flow towards the tank. The tank was placed underneath the PV module.

### Filtration procedure and cleaning process

This cleaning framework pressured water to clean the front surface of the PV module and recycled the water by collecting it through the gutter pipe and returning it to the water tank. The procedure began with the water pump, which transferred water from the tank to the hole-pipe located at the top of the PV module for spraying over the module surface. Before being returned to the tank, the gutter-enclosed pipe and a water filter filtered the dust water. After the completion of the filtration procedure, the recycled water was finally fit for reuse in the subsequent cleaning cycle.

The cleaning process worked at a 15 min interval. Initially, 15 g of dust (wet sand) was spread over the PV surface and dust sensor. Then the PV module was left for 14 min exposed to the sun as no-cleaning process was initiated till this moment. Just at 15min, the dust sensor was turned on by Arduino Uno and it detected dust particles beyond the threshold (0.9$\frac{\mu g}{m^{3}}$). This threshold triggered the pump ON and channeled the water from the reservoir to the PV surface. The cleaning process remained activated for 20 s to perform the cleaning process properly. We recorded PV output data immediately after the cleaning process. We set 40 s intervals between the two cleaning processes for the manual data record. Before the second cleaning process, another 3 g of wet sand was spread over both PV module and continued with the same amount of wet sand repeatedly every 15 min interval until 6:00 PM. The PV module without the cleaning system was just left without cleaning in order to observe the comparison analysis.

The algorithm of the cleaning process was written in C code and burned into the Electrically Erasable Programmable Read-Only Memory (EEROM) of Arduino Uno ATmega328P microcontroller. Arduino Uno controlled the optical dust sensor to sense the dust density near the PV installation place. It also triggered the pump to switch ON and OFF if the threshold of the dust density was exceeded. The algorithm was designed in such a way that Arduino Uno woke up from sleep mode every 15 min and then read the dust density. If the density exceeded 0.9 $\frac{\mu g}{m^{3}}$, it turned the pump on for 20s. The system again went to sleep mode for 15 min.

## Analysis and result discussion

### Simulation and modeling

There are many correlations expressing PV output power, as a function of environmental parameters such as ambient temperature, wind speed, humidity, glazing-cover transmittance, solar irradiance, and module area. Here, we have obtained a model equation of output power, $P_{out}$ relation with such variables shown in Equation 1 [29].

1 $$\begin{aligned} P_{out} = \eta .A.Z.\tau .[1-0.0045(T_{m}-25)] \end{aligned}$$

Here $\eta$, *A*,$\tau$, $T_{m}$, and *Z* refer manufacturer efficiency (%), surface area ($m^{2}$), glazing transmissivity, module temperature (°C), and solar irradiance ($W/m^{2}$). In this equation, we have observed a linear relationship between *Z* and $T_{m}$ in our previous work [28], can be shown in Eq. 2.

2 $$\begin{aligned} Z = 31.82T_{m}-876.51 \end{aligned}$$

Replace *Z* in Eq. (1) by Eq. (2), Equation 3 can be obtained.

3 $$\begin{aligned} P_{out} = \eta .A.[31.82T_{m}-876.51].\tau .[1-0.0045(T_{m}-25)] \end{aligned}$$

By simplifying the above equation, Eq. (4) can be obatined.

4 $$\begin{aligned} P_{out} = \eta .A.\tau .[-0.14319T_{m}^{2}+39.344T_{m}-975.12] \end{aligned}$$

By definition, glazing transmissivity ($\tau$) is inversely proportional to the dust-fall on the PV surface [30]. Since dust-fall can decrease the value of $\tau$, we define it as dust-fall factor, $(1- \tau )$ and empirically we set $\tau =[0.4,0.9]$. A research suggests $\tau =0.9$ for a clean glass based PV surface [31].

**Fig. 5:**
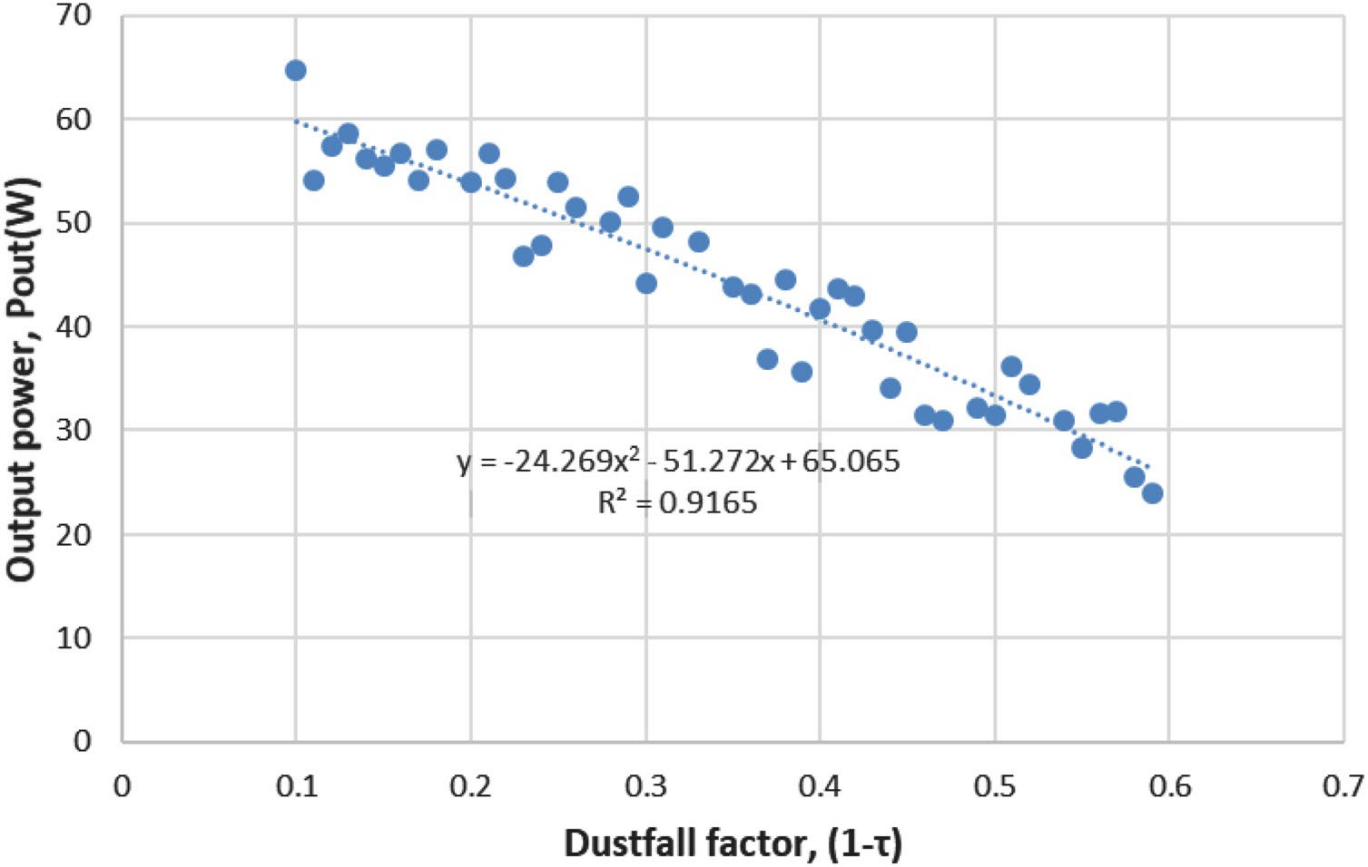
Simulation result—output power ($P_{out}$) against dust-fall factor $(1- \tau )$, a nonlinear relationship between the two parameters

We simulate the model Eq. (4) for a 100W mono type PV module to show the relationship between $P_{out}$ and dust-fall factor, where the constant terms are set from the module specifications as $\eta =0.14$ and $A=0.6583m^{2}$. We consider random values of $T_{m}$ between 50 and 57 °C as per our observation on module temperature range in Malaysia. The simulation result is shown in Fig. 5.

$P_{out}$ and dust-fall factor intensely depend on the weather and PV surrounding conditions. The obtained results support most of the previous work, however some mere discrepancies can be seen due to the climatic parameters of the considered PV location. The presented correlation form is valid for glazing transmissivity ($\tau$) between 40 and 90%. Curve fitting analysis is used to select the optimal regression, whether it is linear, inverse, logarithmic, quadratic, cubic, or exponential, that fits the pattern and expresses the mathematical relationship. Figure 5 displays the best curve-fitting analysis outcomes of Eq. (4). The quadratic interpolation curve fits the data in line with the generated model. The relationship of dust-fall factor and $P_{out}$ are studied based on the coefficient of determination ($R^{2}$) value. $R^{2}$ is obtained as 0.92, which is close to 1 and states the strong negative relation between dust-fall factor and $P_{out}$.

From the result stated in Fig. 5, Eq. (5) can be obtained that relates the dust-fall factor and output power.

5 $$\begin{aligned} P_{out} = -24.269(1-\tau )^{2}-51.272(1-\tau )+ 65.065 \end{aligned}$$

**Fig. 6:**
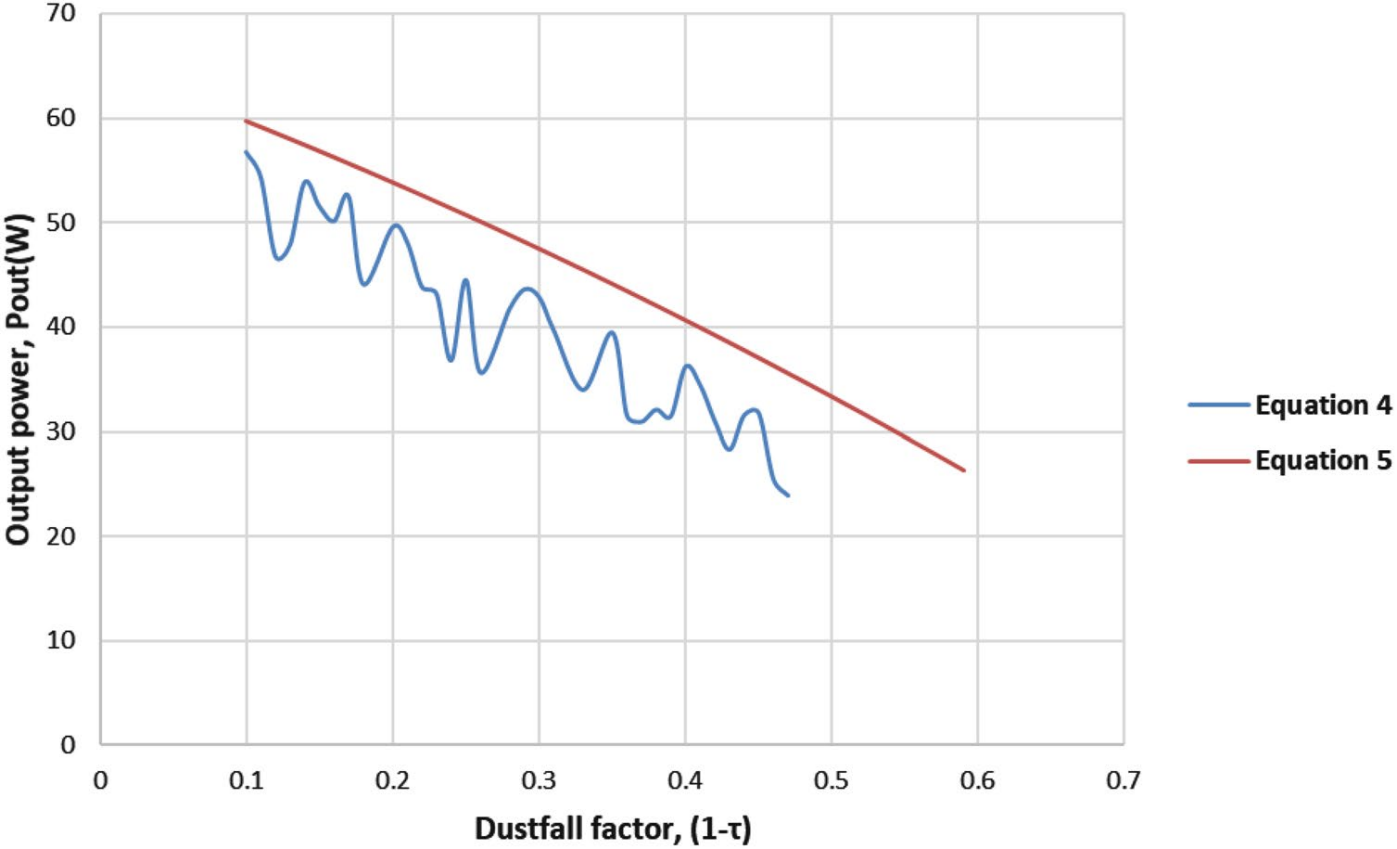
Comparison of $P_{out}$ ratio calculated from Eq. (4) and from quadratic regression Eq. (5) based on simulation

It is necessary to test this equation on the same dataset that was used for the study in order to validate it. For this, we normalize the datasheet and reduce the sample data from 100 to 44. The computed $P_{out}$ obtained from Eq. (4) and the calculated $P_{out}$ obtained from Eq. (5) are compared. The difference is shown in Fig. 6.

Figure 6 demonstrates the comparable patterns in both curves. The range of the difference as a percentage of $P_{out}$ derived using Eq. (4) and the most recent calculated $P_{out}$ by using Eq. (5) is from 0.23% to 14.96% with an average of 6.32%. The statistical error terms, such as mean average percentage error, (MAPE) for Eq. (5) is found to be 6%, whereas root-mean-squared error (RMSE) is 3.02, which shows that the equation is quite acceptable [3]. The overall model summary is shown later in Table 4.

### Experimental data analysis

The obtained experimental data and its analysis have been explained by dividing into two parts. The first section describes the impact of dust on the output power, energy, and efficiency of AWR-based and non-cleaned PV. The results are subsequently compared to manufacturer-recommended parameters and the findings of prior studies. Second, we have analyzed net water loss and the effectiveness of the water recycling process.

We conducted data collection only for one day, from 8.00AM to 6.00PM. The performance of the no-cleaning and AWR method was observed under the cloudy climate. A small amount of wet sand was collected from the nearby surroundings of the PV installation place. Based on our empirical analysis and compared with previous researchers, the approximate size of the dust particles is in the range of 100 to 200 $\mu m$.

#### Electrical parameters analysis

In the analysis, we obtained some parameter values from our previous research [28]. Since it was a rainy and highly cloud-covered day, we considered it as a ’medium luminance day’ with solar irradiance of 340$\frac{W}{m^{2}}$. Similarly, the average module temperature was 40$^oC$, temperature coefficient 0.0045$C^{-1}$. Sun-hour on a medium luminance day was found 3.07*h*. The rest of the values and comparison analysis among the manufacturer specification, experimental data, and other researchers are shown in Table 3.

**Table 3:** Comparative analysis among manufacturer specification, experimental data, and other researchers’ outcomes based on environmental and electrical parameters of no-cleaning and AWR-based PV

Parameters	Units	Data	
		*No- cleaning*	*AWR*
Environmental			
Average solar irradiance$^{*}$	$W/m^2$	340	
Average module temperature$^{*}$	$^oC$	40	
Sun-hour$^{*}$	h	3.07	
Manufacturer Spec			
PV type		Mono	
Maximum power	W	10	
Maximum power voltage	V	20	
Maximum power current	A	0.5	
Efficiency	%	6.6	
Size	$m^2$	$0.54\times 0.28$	
Experimental			
Maximum power	W	3.26	
Maximum power voltage	V	18.09	
Maximum power current	A	0.18	
Average voltage	V	17.51	17.7
Average current	A	0.13	0.16
Average power	W	2.3	2.8
Average energy	Wh	85.60	102.29
Efficiency	%	4.5	5.45

$^{*}$Obtained from our previous research conducted in Malaysia, [28]

Based on the experimental data, we observe that the rate of reduction in the PV module’s performance significantly depends on the rate of dust accumulation on its surface area. Referring to Table 3, we notice that the obtained efficiency for AWR-based PV is 5.45%, which is 20% higher than that of the no-cleaning system. The no-cleaning and AWR methods are 31.82 and 17.42%, respectively less than the manufacturer-rated efficiency. Due to very low luminance, only 25–30% of manufacturer-rated current was generated from the PV module.

**Fig. 7:**
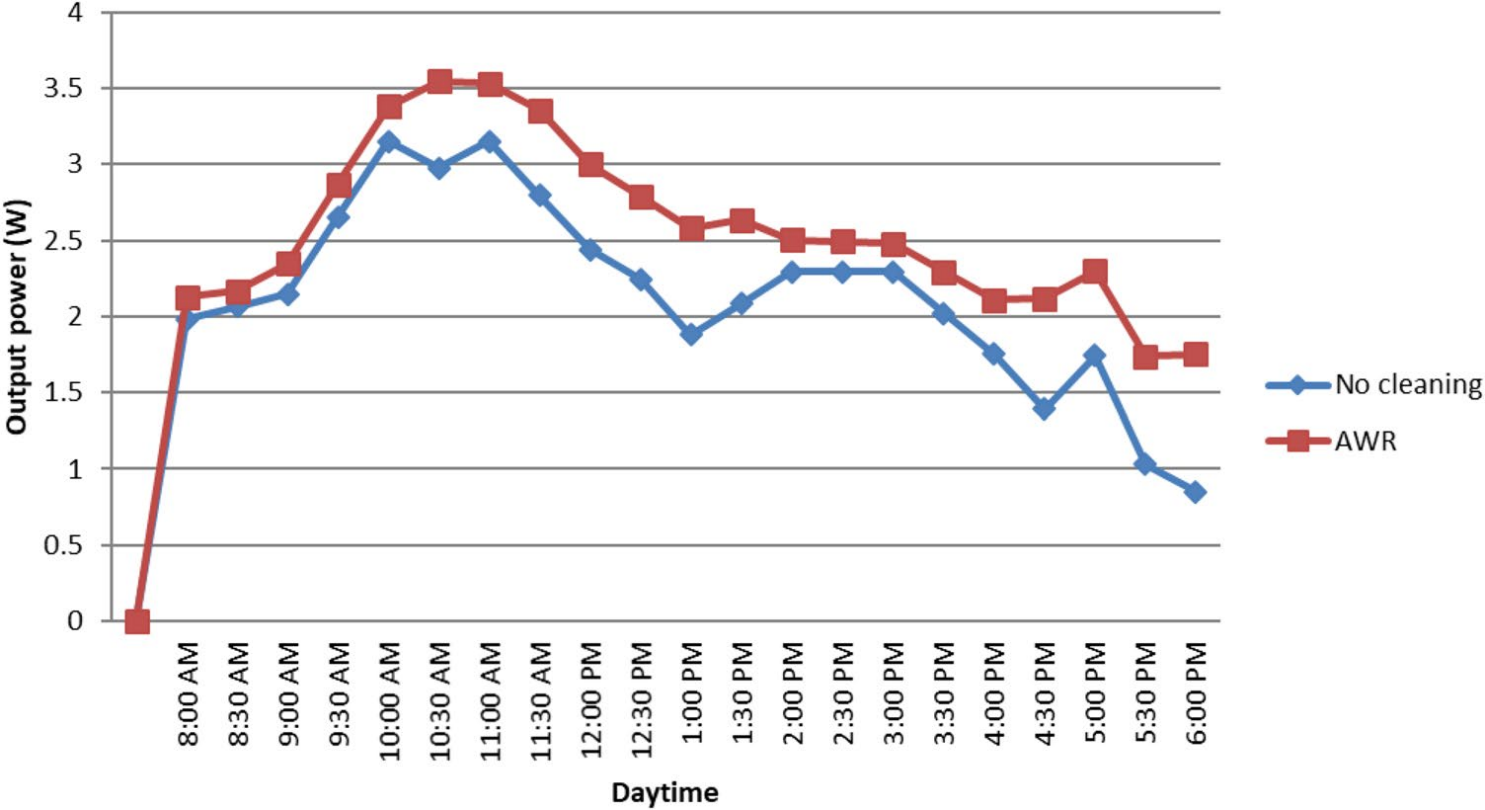
Comparison of the PV output power between AWR method and no-cleaning systems

**Fig. 8:**
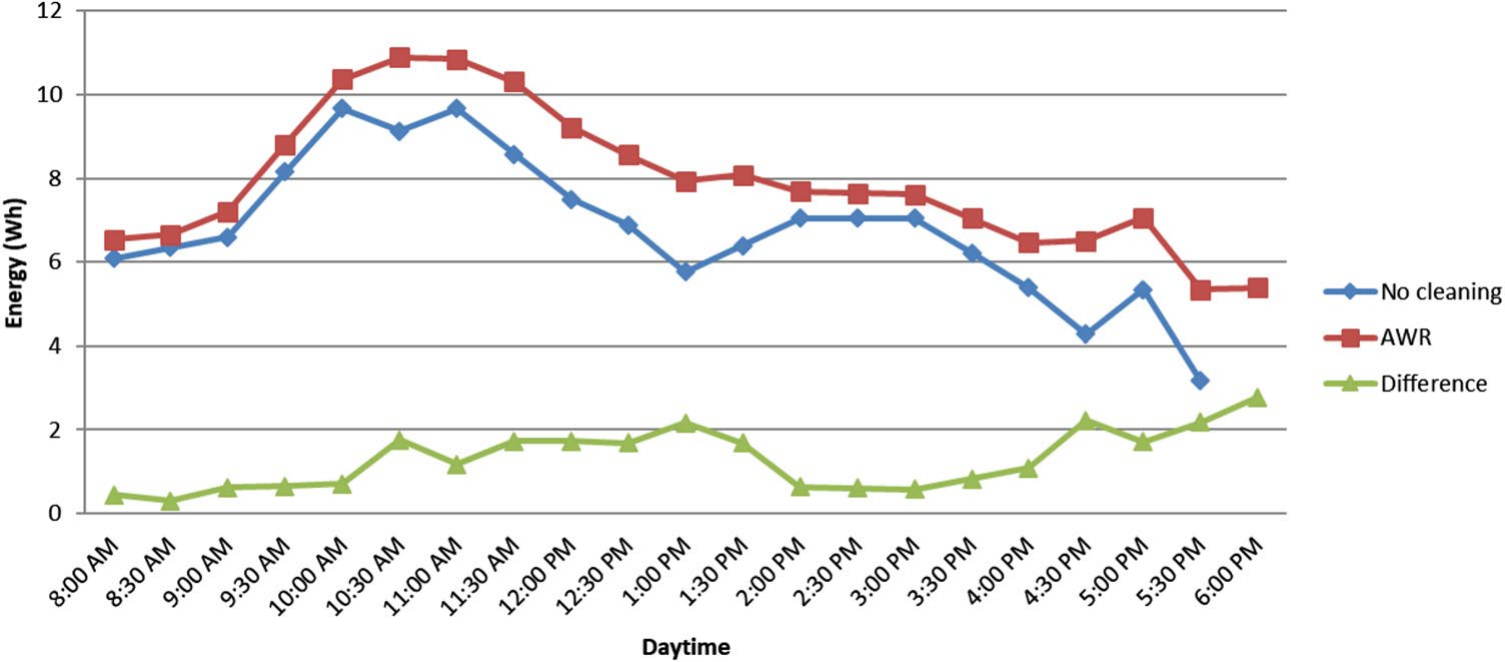
Comparison of the delivered energy and energy difference between AWR method and no-cleaning systems

Figures 7 and 8 show the comparison analysis between no-cleaning and AWR methods. According to Fig. 7, $P_{out}$ from AWR method is 24.40% higher than the no-cleaning system. Initially, we recorded $P_{out}$=3.26W before no sand was applied on both of the PV surfaces. Taking this as the reference of maximum power at 340$\frac{W}{m^{2}}$ irradiance, it is found that 29.44% of loss in $P_{out}$ for the no-cleaning PV module. Almost similar result was found by the researcher from Pakistan − 19.33% power loss for mono type PV module [10].

The energy calculation shown in Fig. 8 is obtained by multiplying sun-hour with $P_{out}$. Dust accumulation reduced $P_{out}$ of the module, which had an adverse effect on the amount of delivered energy.

Figure 8 shows the delivered energy during the test period. As time passed, it was evident that the dusty module’s energy output was lagging behind the clean ones. At the end of the experiment, the total energy output of the AWR and no-cleaning modules were 160.76 Wh and 136.26 Wh, respectively. This also shows an overall improvement of 24.40% in energy yield for the AWR method. This investigation also revealed the variation in energy production is caused by the irradiance throughout the day.

**Fig. 9:**
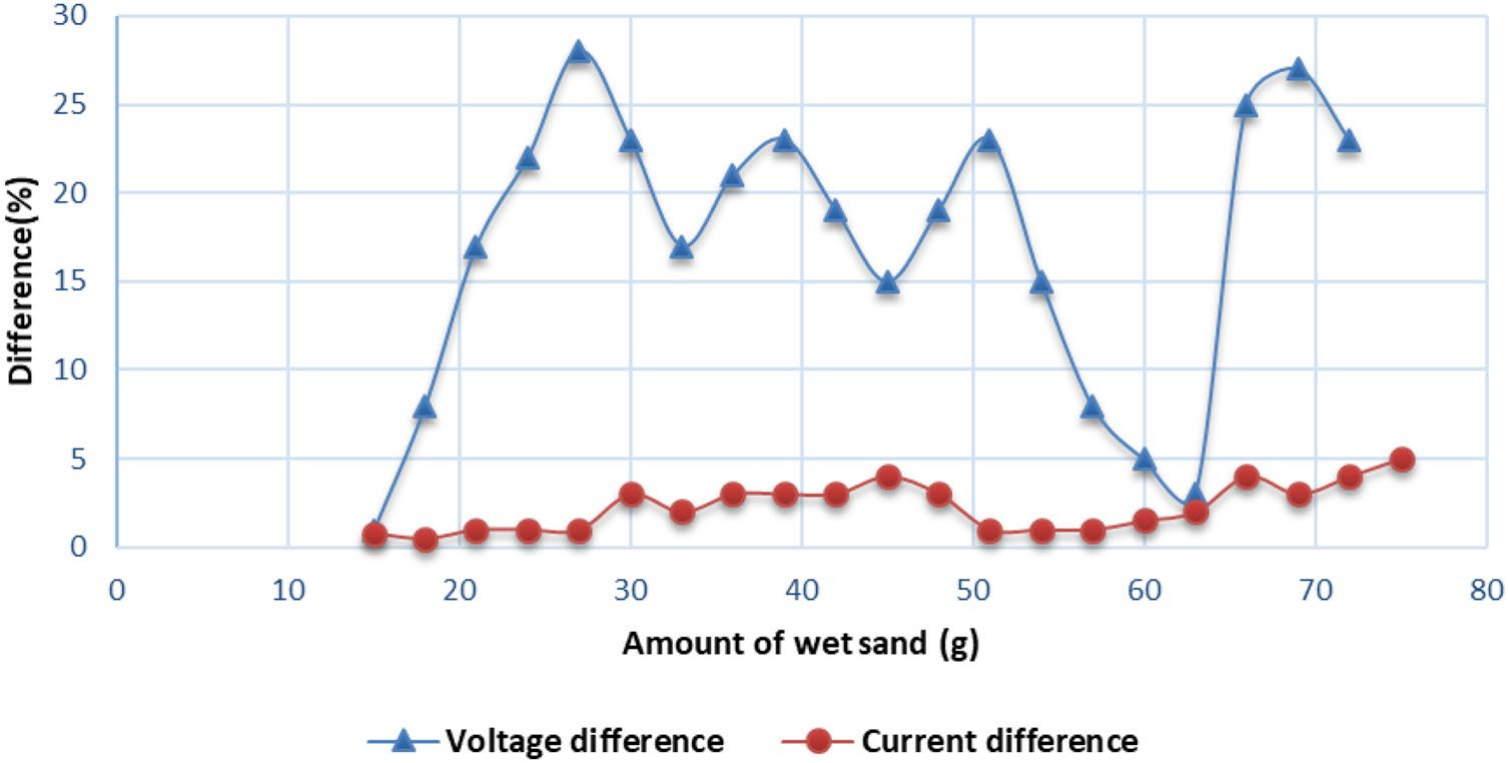
Percentage difference in current and voltage against average amount of wet sand

For a better illustration, the reduction in output voltage as well as the output current with respect to the amount of wet sand are plotted in Fig. 9. It can be observed from Figs. 7 and 8 that the main reason for the reduction in the output power or energy of PV module, due to wet sand accumulation, is the degradation of the output voltage. It can be seen that up to 5 and 28% reduction in output current and voltage, respectively due to wet sand decomposition on the PV surface.

**Fig. 10:**
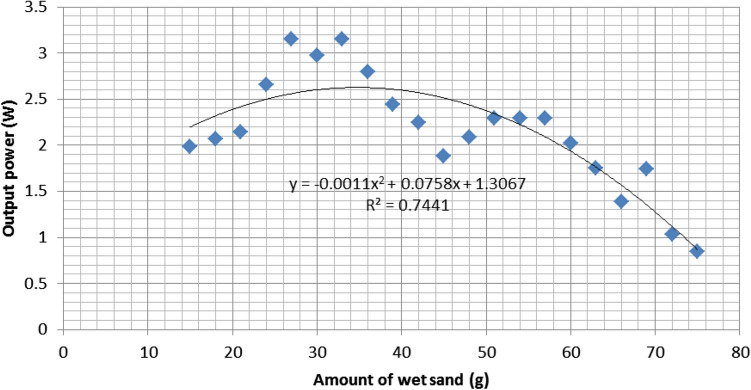
$P_{out}$ vs. amount of wet sand

Figure 10 shows $P_{out}$ against the amount of wet sand. Initially, 15g of wet sand was taken and gradually 3g was added at every 15min over the 0.1512$m^{2}$ surface area. The obtained quadratic equation ($y=-ax^{2}+bx+c$ form) indicates that $P_{out}$ is inversely proportional to wet sand accumulation on the PV surface. Moreover, the optimum formula for $P_{out}$ can be modeled in Eq. (6). The accuracy of this model equation can be further demonstrated by the $R^{2}=0.744$, which is moderately strong. The model equation is based on the regression analysis.

6 $$\begin{aligned} P_{out} = -0.0011D_{m}^{2}+0.075D_{m}+1.3067 \end{aligned}$$

Here, $D_{m}$ is the amount of wet sand (g). $-0.0011D_{m}^{2}$ indicates a negative correlation between dust accumulation and $P_{out}$. This relation supports the model (Eq. 5) obtained in the simulation analysis.

**Fig. 11:**
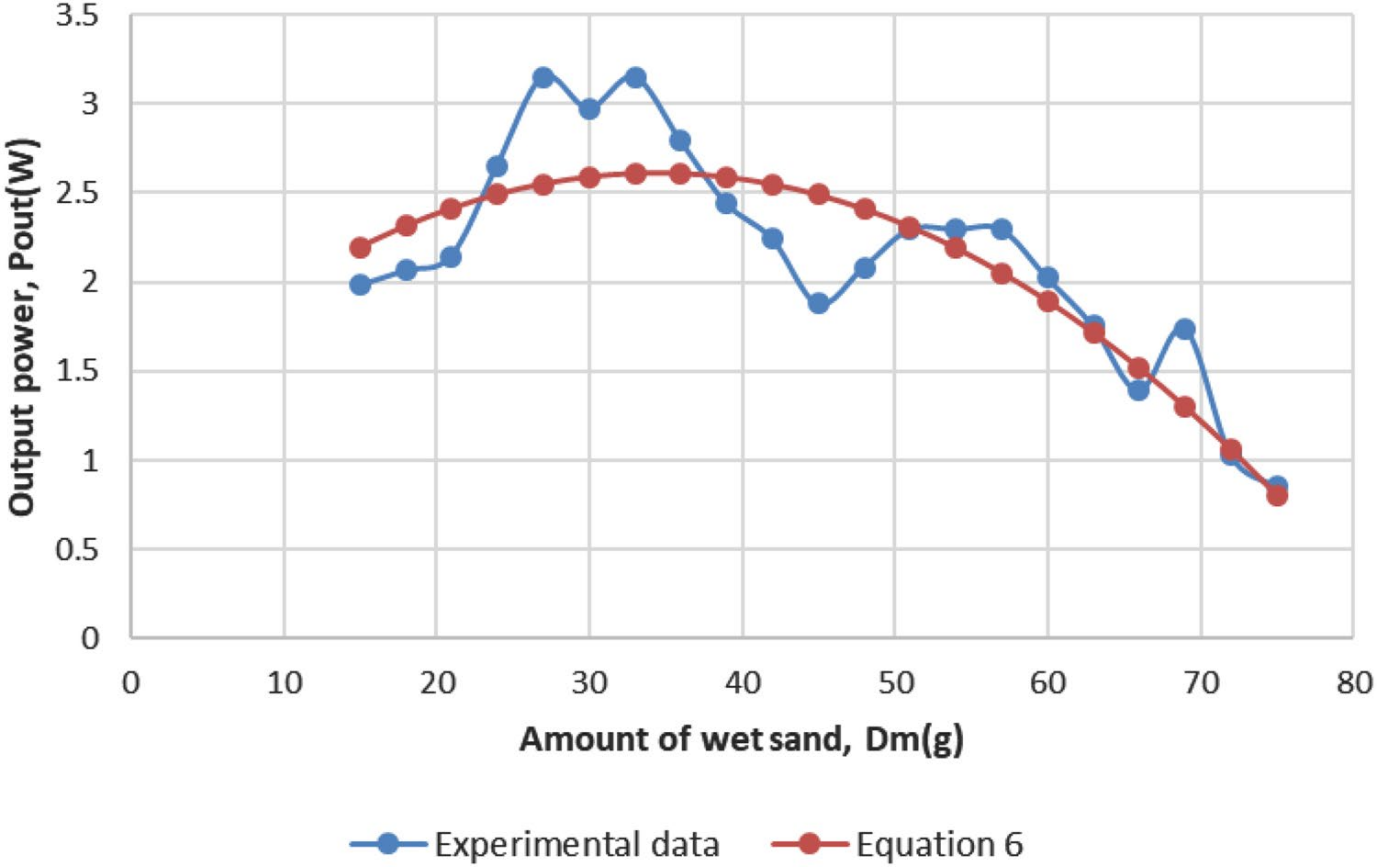
Comparison of $P_{out}$ ratio calculated from experimental data and from quadratic regression Eq. (6)

Now, similar to the analysis explained in simulation section (Sect. 4.1), Eq. (6) can be validated by testing it on the experimental data. The comparison is depicted on the calculated $P_{out}$ obtained from experimental data and the calculated $P_{out}$ obtained from Eq. (6). Figure 11 shows the comparison. Similar behavior of $P_{out}$ versus dust deposition can be found in other studies such as [30].

We observe both curves in Fig. 11 showing the similar trends. The range of the difference as a percentage of $P_{out}$ obtained from experimental data and the latest calculated $P_{out}$ by using Eq. (6) is from 0.007 to 0.29% with an average of 0.11%. The statistical error terms, such as MAPE for Eq. (6) is found to be 11%, whereas RMSE is 0.30, which shows that the equation is very acceptable.

We compare the statistical terms obtained from our simulation analysis, experimental data, and previous researchers’ outcomes. The comparison of the model summary is shown in Table 4. We observe that the regression type of both simulation and experimental is quadratic, where the other statistical terms are quite similar. Least value of systematic error terms, such as Std. error of estimate, MAD, MSE, RMSE, and MAPE are desirable and it is achieved with acceptable estimation for both models. This validates both of the model equations. The accuracy of the models can be demonstrated by the $R^{2}$ value outstandingly in simulation analysis (Fig. 6) compared with the model from experimental data (Fig. 11), which are moderate. The high value of $R^{2}$, 0.92 and 0.74 from simulation and experimental analysis respectively, implies that there is a significant relationship between $P_{out}$ and dust-fall factor. However, the model from experimental data outperforms the simulation model for systematic error analysis, which means that it is more accurate than that of the simulation model. For further validation, the obtained results are also compared with the previous researchers’ outcomes and found to be in-line with their results.

**Table 4:** Comparison of curve fitting statistical analysis for $P_{out}$ and dust-fall factor data regression among simulation analysis, experimental data, and previous researchers’ outcomes. The systematic error terms, namely MAD, MSE, RMSE, and MAPE refer mean absolute deviation, mean square error, root mean square error, and mean absolute percentage error. NA denotes not available

Statistical terms	Simulation	Experimental	Previous studies
Regression type	Quadratic	Quadratic	Cubic$^{\dag }$; polynomial power 6$^{\ddag }$; exponential$^{\P }$
$R^{2}$, adjusted $R^{2}$	0.92, 0.91	0.74, 0.710	0.92, 0.91$^{\dag }$; 0.99, 0.99$^{\ddag }$; 0.92, NA$^{\P }$
Std. error of estimate	0.47	0.31	0.21$^{\dag }$
Percentage difference (%): *(Min, Max, Avg)*	0.23, 14.96, 6.32	0.0073, 0.29, 0.11	1.68, 8.79, 6$^{\dag }$; NA,6.85,NA$^{\P }$
MAD	2.59	0.25	NA$^{\dag }$ $^{\ddag }$ $^{\P }$
MSE, RMSE	9.17, 3.03	0.09, 0.30	1.21, 1.10$^{\dag }$; 0.16, 0.40$^{\ddag }$; 0.0002, 0.013$^{\P }$
MAPE	0.06	0.11	0.05$^{\dag }$

$$^{\dag }$$ [3];

$$^{\ddag }$$ [32];

$$^{\P }$$ [30]

The scenario from this study indicates that, in this rooftop based NEM application, dust-fall can be scattered widely on the PV surface carried by the wind. Over time, the effect of dust-fall can be severe if it is not cleaned regularly. Even though the calculated results from either Eqs. (5) or (6) is not practically 100% accurate, it is still able to produce values near to the real data within the imprecision $<15$%. Hence, it still can be used to forecast $P_{out}$ of PV modules on this site. In addition, the forecasting can be much simpler by using either of the equations compared to the other models. Meanwhile, the developed either of the models is able to predict $P_{out}$ in this rooftop application by using the only known dust-fall on the PV surface. On the other hand, soiling estimation through the relationship of $P_{out}$ and dust-fall factor or amount is still absent under the climate of Malaysia from the published research literature. So by having the dust amount in this region and using either of the equations, $P_{out}$ of the PV module can be calculated and necessary actions for the design and optimization of systems can be performed.

#### Water recycling process and net loss

**Fig. 12:**
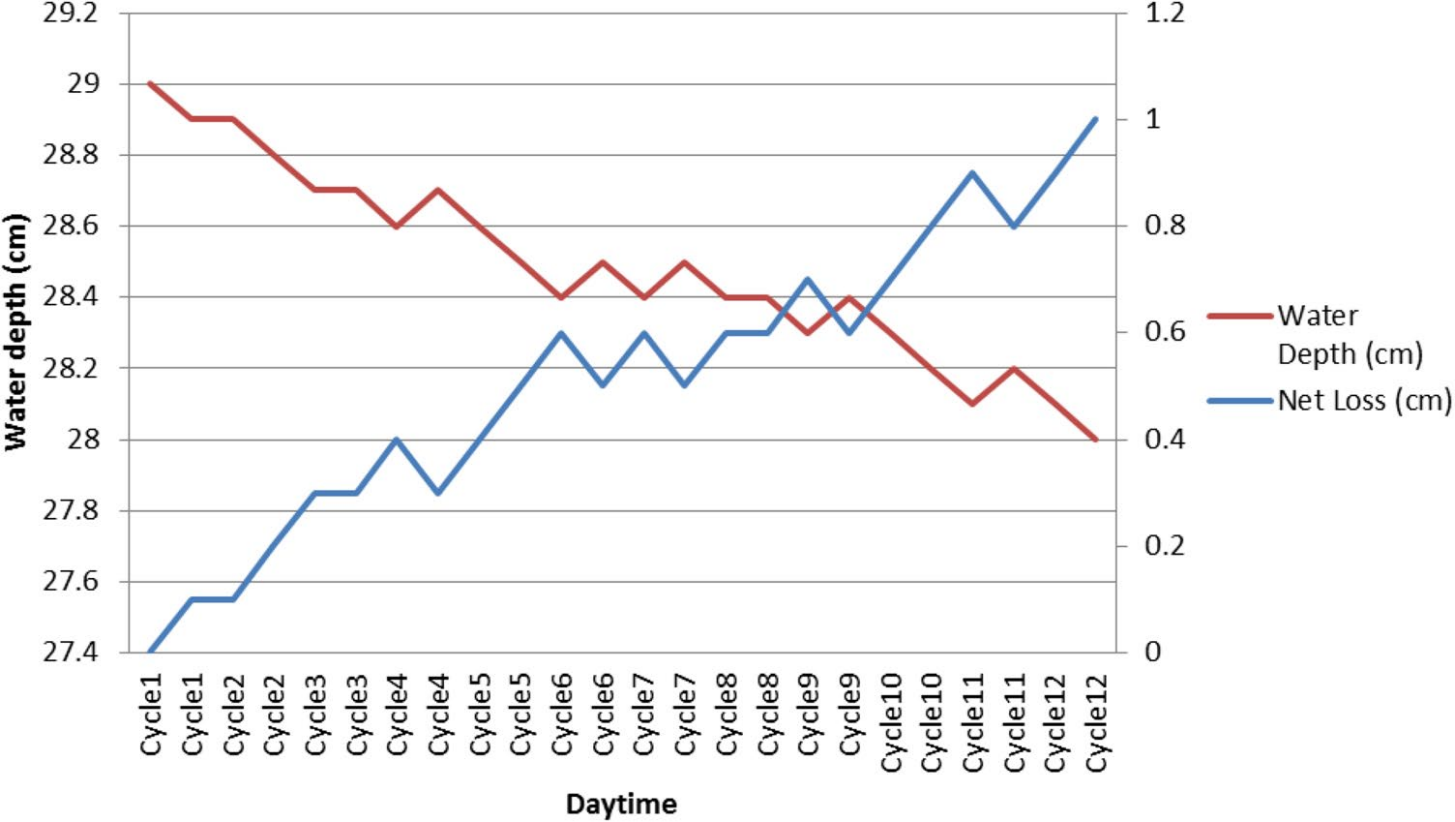
water recycling process analysis. Water depth indicates the height of the water in the tank

In the experiment, the 15*L* capacity of the cylinder shape reservoir tank (diameter= 0.24*m*, height of maximum water level = 0.29*m*) was filled up with 13.1*L* of water. This amount of water was reached till 29*cm* (or 0.29*m*) height of the tank. Initially, the net water loss was 0.0*L*, but over the time it increased due to water droplets sprayed through the holes and evaporation. Each cycle requires 20*s* to complete the cleaning process. Both water depth and net loss against cleaning cycle are shown in Fig. 12. The overall net water loss was found 0.5*L* (water level decreases to 1cm) or 3.83% for the 12 cycles of cleaning. In order to validate our result with the previous researcher, we calculated the loss/cycle of the proposed AWR method, which can be computed at 0.32%/cycle. If the cleaning process is conducted for 90 days at frequency of 1 cycle/day, the net loss can be obtained 28.8%. This result is better than [10] where they obtained 55% of water recycling capacity from the 90 days’ experiment. This yields AWR as an effective cleaning method.

Since the water recycling system in PV cleaning has a great effect on minimizing the water consumption, it can be applied in NEM applications with a mere change in any existing household water supply system. In the hot and dry regions, water crisis is always a crucial issue, thus a water recycling system can be a useful solution to reduce water loss up to 28.8% in 90 days’ cleaning operation. Further, it is not essential to perform water spray for cleaning all the year round as we observed from our dust density analysis in Fig. 2. It can be utilized only during the dry season or during the months of less rainfall. Rain sensor together with an AWR system can be an effective solution to reduce more water loss.

## Conclusion and recommendations

Wet sand adhesion on the PV surface in a fully humid tropical country like Malaysia is identified as one of major causes of the module to operate less efficiently. Considering this fact, the idea is to investigate AWR or automated water recycle method for dust cleaning in residential rooftop based NEM applications. In the simulation, we have developed a new mathematical model, which is much simpler and accurate to predict output power, $P_{out}$ with only known dust-fall factor on the PV surface. In the model analysis, dust-fall factor is found to be significant ($R^{2}=0.92$) for $P_{out}$ reduction, and from this, a new mathematical model has been regressed and developed. The new quadratic model deviates from the previous used model (Eq. 4) from 0.23 to 14.96% with an average of 6.32% when comparing the accuracy of forecasts using the statistical data. The calculated MAPE for this model is found at 6%, whereas RMSE at 3.02 shows that the equation is acceptable and applicable for the researched site. To validate the model, we have implemented the recycling system using a low-powered DC pump controlled by the programmable microcontroller. Then we have tested the system taking two identical 10W mono type PV module installed at 15 °C tilt angle and investigated $P_{out}$ variation on a medium luminance day ($340\frac{W}{m^{2}}$) affected by the wet dust adhesion.

The result of this investigation shows 24.40% higher in $P_{out}$ than that of a no-cleaning system. The study finds that degradation in output voltage due to the wet sand decomposition on the PV surface is the main reason for the reduction in the output power or energy of the PV module. It is observed that about 5% and 28% reduction in output current and voltage cause approximately 29.44% loss in $P_{out}$ for a no-cleaning system. Another major finding is that the efficiency of the PV module can be achieved up to 5.45% after the deployment of the AWR method. The obtained efficiency is 20% more than that of the no-cleaning system.

The loss per cycle of the AWR method is computed only 0.32%/cycle which yields 28.8% of total water loss in 90 days’ cleaning operation. In the investigation, the overall net water loss is found to be 3.83% from the 12 cycles of PV surface cleaning. The aftermath of the process has outperformed the previous studies.

Finally, we have obtained another new quadratic model in the same research site from the experimental data to forecast $P_{out}$ with just one variable—amount of dust-fall. The regression analysis of the model reveals a negative relationship between $P_{out}$ and amount of dust-fall. Although $R^{2} (=0.74)$ of the experimental model is slightly lesser than the simulation model, statistical terms confirm its better accuracy from the analysis of std. error of estimate (0.31), percentage difference (0.0073-0.11), MAD (0.25), and RMSE (0.30). Both models are simplifications of the wet soiling prediction performed under the climate of Malaysia.

The AWR method is recommended for the rooftop PV system. Adding dispersants into the water can help to break down the sticky dust, such as birds’ droppings into smaller droplets and do a more effective cleaning. A key policy priority should therefore be to plan for the whole year investigation of total energy yield in kWh of the PV system to come up with a decision of integrating the AWR cleaning method. The proposed cleaning system can be recommended to test for at least a year, especially the dry season in Malaysia to investigate a faster payback period of NEM investment.

## Data Availability

The data that support the fndings of this study are available from the corresponding author, upon reasonable request.
